# Selenoprotein S (SEPS1) gene -105G>A promoter polymorphism influences the susceptibility to gastric cancer in the Japanese population

**DOI:** 10.1186/1471-230X-9-2

**Published:** 2009-01-13

**Authors:** Tomoyuki Shibata, Tomiyasu Arisawa, Tomomitsu Tahara, Masaaki Ohkubo, Daisuke Yoshioka, Naoko Maruyama, Hiroshi Fujita, Yoshio Kamiya, Masakatsu Nakamura, Mitsuo Nagasaka, Masami Iwata, Kazuya Takahama, Makoto Watanabe, Ichiro Hirata

**Affiliations:** 1 Department of Gastroenterology, Fujita Health University, School of Medicine, Toyoake, Japan

## Abstract

**Background:**

Inflammation is a key factor in the process of carcinogenesis from chronic gastritis induced by *Helicobacter pylori*. Selenoprotein S (SEPS1) is involved in the control of the inflammatory response in the endoplasmic reticulum (ER). Recently the -105G>A polymorphism in the promoter of SEPS1 was shown to increase pro-inflammatory cytokine expression. We examined the association between this polymorphism and the risk of gastric cancer.

**Methods:**

We took stomach biopsies during endoscopies of 268 Japanese gastric cancer patients (193 males and 75 females, average age 65.3), and 306 control patients (184 males and 122 females, average age 62.7) and extracted the DNA from the biopsy specimens. All subjects provided written informed consent. For the genotyping of the SEPS1 promoter polymorphism at position -105G>A, PCR-RFLP methods were used and the PCR products were digested with PspGI.

Logistic-regression analysis was used to estimate odds ratios (OR) and 95% confidence intervals (CI), adjusting for age, sex, and *H. pylori *infection status.

**Results:**

Among cases, the distribution of genotypes was as follows: 88.4% were GG, 11.2% were GA, and 0.4% were AA. Among controls, the distribution was as follows: 92.5% were GG, 7.2% were GA, and 0.3% were AA. Among males, carrying the A allele was associated with an increased odds of gastric cancer, compared with the GG genotype (OR: 2.0, 95% CI 1.0–4.1, p = 0.07). Compared with the GG genotype, carrying the A allele was significantly associated with increased risks of intestinal type gastric cancer (OR: 2.0, 95%CI 1.0–3.9, p < 0.05) as well as of gastric cancer located in the middle third of the stomach (OR: 2.0, 95%CI 1.0–3.9, p < 0.05).

**Conclusion:**

The -105G>A promoter polymorphism of SEPS1 was associated with the intestinal type of gastric cancer. This polymorphism may influence the inflammatory conditions of gastric mucosa. Larger population-based studies are needed for clarifying the relation between inflammatory responses and SEPS1 polymorphism.

## Background

Gastric cancer remains a considerable public health problem worldwide. Although the incidence and mortality rates of gastric cancer have decreased gradually, gastric cancer is second only to lung cancer as the leading cause of cancer death around the world [1,2]. *Helicobacter pylori *(*H. pylori*) was designated as a causative pathogen for gastric carcinogenesis[3]. Inflammation may be a key factor in the process of carcinogenesis from chronic gastritis induced by *H. pylori*[4]. However, only a small number of infected patients actually develop gastric cancer. This suggests that host genetic factors, such as genes associated with inflammatory responses, may also play an important role in stomach carcinogenesis.

Selenoprotein S (SEPS1, also known as SelS, SELENOS, VIMP) is a novel selenoprotein located in the endoplasmic reticulum (ER) and the plasma membrane. It is involved in the control of the inflammatory response in ER[5].

SEPS1 protects cells from oxidative damage and apoptosis, and is widely expressed in a variety of tissues [6-8].

Recently, the -105G>A promoter polymorphism of SEPS1 was shown to be strongly associated with plasma levels of pro-inflammatory cytokines, such as interleukin 1 beta (IL-1β), interleukin 6 (IL-6) and tumor necrosis factor alpha (TNF-α). The -105G>A promoter polymorphism of SEPS1 may affect SEPS1 mRNA levels[9]. The substitution of the A allele for the G allele at position -105 reduced the promoter activity in HepG2 cells[10].

After the report of a polymorphism in the SEPS1 promoter region, some clinical studies have shown associations between the SEPS1 polymorphism with coronary heart disease and ischemic stroke[11] and preeclampsia[12], while other studies reported no associations between this variant with inflammatory bowel disease[13] or cerebrovascular disease[14].

However, the association between the -105G>A promoter polymorphism of SEPS1 and gastric cancer risk has not yet been studied.

IL-1β levels have been associated with gastric cancer[15], and because the SEPS1 polymorphism affects the levels of IL-1β, the SEPS1 polymorphism may be an important genetic factor for the development of gastric cancer.

In this case-control study, we examined the associations between the SEPS1 promoter polymorphism and both the gastric cancer risk and the inflammatory responses in gastric mucosa as measured in gastric biopsy samples.

## Methods

### Patients and Controls

The study included patients who received an upper gastrointestinal endoscopy at the Fujita Health University Hospital in Japan. Consecutively enrolled subjects were screened for gastric cancer by upper gastrointestinal endoscopy followed by a barium X-ray examination. Cases were 268 Japanese patients (193 males, 75 females, average age 65.3 ± 12.0 years) who were diagnosed with primary gastric cancer, and controls were 306 people (184 males, 122 females) without gastric cancer who also underwent upper gastrointestinal endoscopy. Gastric cancer was diagnosed histologically at the Pathology Division of our hospital, and cancers was classified according to Lauren' s classification[16]. The cancer staging and anatomic location information were also obtained. Patients with systemic diseases and malignancies in other organs, or who had received non-steroidal anti-inflammatory drugs were excluded.

The Ethical Committee of the School of Medicine at Fujita Health University approved the protocol. Written informed consent was obtained from each study participant.

### Histological study and DNA Extraction

Gastric biopsy specimens were taken from the non-cancerous mucosa in the antrum and the greater curvature of the stomach, using an upper gastrointestinal scope. Some parts of each specimen were fixed in 10% buffered formalin and embedded in paraffin, while the other parts were immediately frozen and stored at -80°C until DNA extraction. All histological diagnoses were made at the Division of Pathology of our hospital. The severity of chronic gastritis was classified by a pathologist who did not have access to any clinical information, according to the updated Sydney system[17] with each factor being scored from 0 (normal) to 3 (marked).

The genomic DNA was extracted from stored samples at -80°C using proteinase K and DNA extraction kits (Quiagen, Valencia, CA).

### Polymorphism Analysis of SEPS1 Gene

The genotype for the SEPS1 promoter region at position -105 was determined using PCR-based restriction fragment length polymorphisms (PCR-RFLP). We used primers that included the SEPS1 (-105) polymorphism area. Subsequently, the identification was done after PCR-amplification, using the following primers 5'-AAATCCGTGAACGAGGTCCG-3'and 5'-GAGCAACTAATCTGAATCAGG-3'. PCR was carried out with 0.1 μg of genomic DNA in a volume of 20 μL. The DNA was denatured at 94°C for 5 minutes, followed by 35 cycles at 94°C for 20 seconds, 53°C for 20 seconds, and 72°C for 40 seconds, with a final extension at 72°C for 7 minutes. The PCR reactions were done using Blend Taq (Toyobo Co., Ltd., Osaka, Japan). The amplified PCR products were digested overnight with 5 units of PspGI (New England BioLabs, Inc., Beverly, MA, USA) at 75°C. Subsequently, the digested products were analyzed on 3% agarose gels. These gels were stained with ethidium bromide (0.5 μg/mL), and the genotypes were determined by analyses of different bands. The presence of a PspGI site was indicated by the cleavage of the 324 bp amplified product to yield fragments of 179 and 145 bp. Genotyping was confirmed by direct sequencing in a few randomly selected samples.

### Detection of H. pylori

*H. pylori *positivity was determined by microscopic examination, urea breath test, or serum anti-HP antibody titers. Infection was diagnosed when at least one of these tests was positive.

### Statistical Analysis

Hardy-Weinberg equilibrium of the SEPS1 gene allele in the controls and gastric cancer patients were assessed by χ^2 ^statistics. Clinical characteristics between patients with or without gastric cancer, and differences in gastritis scores between A carriers and G/G were examined by the Mann-Whitney U test. Logistic-regression analysis was used to estimate odds ratios (OR) and 95% confidence intervals (CI) for the genotypes, with adjustment for age, sex, and *H. pylori *infection status. A p-value < 0.05 was considered statistically significant.

## Results

### Characteristics of the subjects

The characteristics of the cases and controls are summarized in Table 1. *H. pylori *infection and age were higher in gastric cancer patients than in controls (p < 0.05).

**Table 1:** Characteristics of subjects

	GC	Controls	*P*
number	268	306	
males/females	193/75	184/122	N.S.
Average age (± SD)	65.3 ± 12.0	62.7 ± 13.2	< 0.05^a^
HP positive rate (%)	86.2	68.0	< 0.05^a^

GC: gastric cancer, HP: *Helicobacter pylori*

^a^GC vs. Controls, Mann-Whitney U test.

The main endoscopic findings for the control group were: gastric ulcer in 68 patients (22.2%), duodenal ulcer in 35 (11.4%), gastric and duodenal ulcer in 5 (1.7%), and gastritis in 198(64.7%).

### Distribution of the SEPS1 genotypes

Table 2 shows the genotype frequencies of SEPS1 in patients with gastric cancer and the control group. The polymorphism at position -105 of SEPS1 was typed in all 574 subjects. Among cases, the distribution of genotypes was as follows: 88.4% were GG, 11.2% were GA, and 0.4% were AA. Among controls, the distribution was as follows: 92.5% were GG, 7.2% were GA, and 0.3% were AA (Table 2). The frequency of SEPS1 polymorphism in the controls and gastric cancer patients did not deviate significantly from those expected under the Hardy-Weinberg equilibrium (p = 0.42, 0.96 respectively). Among males, compared with the GG genotype, the genotypes GA and AA combined was associated with an increased odds of gastric cancer (OR: 1.97, 95% CI 0.95–4.06, p = 0.067; Table 3).

**Table 2:** SEPS1 polymorphism and GC risk

genotypes	patients with GC n (%)	control patients n (%)	OR (95%CI)	*P*
G/G	237 (88.4)	283 (92.5)	reference	
G/A	30 (11.2)	22 (7.2)	1.66 (0.91–3.01)	0.097
A/A	1 (0.4)	1 (0.3)	0.77 (0.05–12.44)	0.852
G/A+A/A	31	23	1.61 (0.90–2.89)	0.112

GC: gastric cancer, CI: confident interval

**Table 3:** Association between SEPS1 polymorphism and gender

		genotype			G/G vs. G/A+A/A	
		G/G	G/A	A/A	OR (98%CI)	*P*

Control patients	male	171	12	1	reference	
	female	112	10	0		
GC patients	male	169	23	1	1.97(0.95–4.06)	0.067
	female	68	7	0	1.17(0.42–3.23)	0.764

GC: gastric cancer, CI: confident interval

In additional analyses, the associations between the SEPS1 polymorphism and clinicopathologic features of gastric cancer, such as the tumor location, stage, and Lauren's histological classification, were evaluated. Carrying a -105 A allele was significantly associated with increased risks of Lauren's intestinal type of gastric cancer (OR: 1.99, 95%CI 1.01–3.93, p < 0.05) and of gastric cancer located in middle third part of the stomach (OR: 2.01, 95%CI 1.03–3.92, p < 0.05) (Table 4).

**Table 4:** Association between SEPS1 polymorphism and tumor location, staging and Lauren's classification

Variables(n)	genotype			G/G vs. G/A+A/A	
	G/G	G/A	A/A	OR(95%CI)	*P*

Patients without GC(306)	283	22	1	Reference	
					
Tumor location					
Cardia(5)	5	0	0	1.93(0.40–9.22)	0.411
Non-cardia(263)	232	30	1	2.81(0.69–11.48)	0.15
Upper third(16)	14	2	0	ND	
Middle third(134)	115	19	0	2.01(1.03–3.92)	0.041
Lower third(113)	103	9	1	1.18(0.52–2.64)	0.696
					
Staging					
Early(138)	121	17	0	1.74(0.88–3.47)	0.112
Advanced(130)	117	12	1	1.52(0.74–3.12)	0.253
					
Lauren's classification					
Intestinal type(151)	132	19	0	1.99(1.01–3.93)	0.047
Diffuse type(110)	100	9	1	1.19(0.54–2.63)	0.659
Mixed(7)	5	2	0	6.82(1.07–43.65)	0.043

NOTE: All data are adjusted for sex, age, and *H. pylori*infection status. ND: not determined, GC: gastric cancer, CI: confident interval

Among 301 *H. pylori*-positive subjects, the neutrophil infiltration, mononuclear cell infiltration, the atrophy and metaplasia scores of the antral mucosa showed no significant differences between the -105 A allele carriers and subjects homozygous for the G allele (data not shown). Among controls, there were no significant genotype differences among the patients with gastric ulcer, duodenal ulcer, and gastritis (data not shown).

## Discussion

In this study of a Japanese population, we have demonstrated for the first time that the -105G>A polymorphism of SEPS1 gene was associated with increased risks of intestinal type of gastric cancer and gastric cancer located in middle third part of the stomach. Although low selenium status has been associated with risk of human gastric cancer [18,19], and high selenium diet inhibit the growth of *H. pylori *in Guinea pigs [20], human clinical trials have failed to demonstrate a benefit for selenium supplements in the prevention of precancerous gastric lesions [21]. The -105G>A promoter polymorphism, which is located in an ER stress response element of the seps1 gene coding for SEPS1, was strongly associated with circulating levels of pro-inflammatory cytokines, such as IL-1b, IL-6, and TNF-a, and with SEPS1 gene expression levels in humans[9]. The phenotypic consequences of this -105G>A polymorphism have been investigated in some diseases related to chronic inflammation [11-14]. The results have been inconsistent. One study in Finnish cohort showed the relation of other SEPS1 SNPs and coronary heart disease or ischemic stroke event[11], and one report in a large Norwegian case-control cohort showed the A allele of the SEPS1-105G>A polymorphism is a significant risk factor for preeclampsia[12]. On the other hand, one report in Germany showed the SELS-105G>A polymorphism was not associated with IBD susceptibility and did not contribute to a certain disease phenotype or increased TNF-alpha levels in IBD patients[13]. Also one report showed non-significant differences of SEPS1 allele frequencies between young stroke patients and healthy controls from Italy and Germany[14].

In our study, there was no association overall between the -105G>A variant and gastric cancer. However, in detailed clinicopathological analysis, we found significant associations between carrying the A allele and the odds of specific types of gastric cancer, with adjustment for age, sex, and *H. pylori *infection status. We have 74 healthy volunteer DNA (all are *H. pylori *negative). We examined these DNA about SEPS1 polymorphism -105G>A. As a result, the numbers of GG were 70, the numbers of GA were 4 and none of them was AA. From these results, we thought our comparison between gastric cancer patients and non gastric cancer patients was proper comparison for analyzing the association risk of gastric cancer and SEPS1 polymorphism other than the association of *H. pylori*.

The carcinogenic pathway for the intestinal type of gastric cancer mainly involves *H. pylori *infection. The infection causes inflammation and tissue regeneration, and these processes cause the deviation from the normal pathway of gastric differentiation to precancerous states[4,22]. It has been suggested that the SEPS1 polymorphism may be involved in the pathway from chronic gastric inflammation to carcinogenesis. However, the AA genotype is very rare in the Japanese population, and thus, definite conclusions about the genotype-phenotype correlations of homozygous carriers of this SEPS1 promoter polymorphism can only be drawn from larger studies involving multiple hospital centers.

Among males, the SEPS1 -105A allele carriers had increased odds of gastric cancer compared with those with the GG genotype. There is one report showing gender-specific associations of the SEPS1 polymorphism with coronary heart disease[11]. The authors of the study found relations between a polymorphism in another region of the SEPS1 gene and coronary heart disease risk in females. They suspected that the gender-specific associations with SEPS1 SNP were due to differences in disease etiology or in the hormonal milieu for men and women [11]. In epidemiological studies, gender has also been related to gastric cancer [23,24]. These epidemiological findings are consistent with the gender specific association between the -105G>A polymorphism and gastric cancer.

Based on the reports of the consequences of impaired SEPS1 gene expression and of the SEPS1 -105G>A polymorphism being functionally involved in inflammatory responses[9], we further assessed gastritis scores in the non-cancerous areas of the antrum in H. pylori positive patients. However, we did not find significant differences between SEPS1 -105GG homozygotes and -105A allele carriers (data not shown). It is possible that the number of cases for this analysis were relatively low. Also, this investigation was not a direct comparison of inflammatory cytokine levels by genotypes of this polymorphism as has been reported in the literature[9]. Therefore, the exact role of selenoproteins in gastric carcinogenesis and inflammation has not yet been determined. Previous reports have found associations between cytokines and gastric cancer[15,25]. In future studies, the analysis of these inflammatory cytokines in gastric cancer will be useful for understanding the mechanisms of carcinogenesis in the stomach.

## Conclusion

In conclusion, carrying an A allele at the SEPS1 -105G>A polymorphism is a risk factor for the intestinal type of gastric cancer and gastric cancer located in the middle third of the stomach in a Japanese population. The A allele may be contributing to gastric cancer by influencing the inflammatory response to *H. pylori *infection in the stomach. Further studies in larger cohorts of gastric cancer may be needed to investigate the actual role of SEPS1 and the important implications of genetic alterations of the selenoproteins in chronic inflammatory responses and carcinogenesis in the stomach.

## Competing interests

The authors declare that they have no competing interests.

## Authors' contributions

TS, TT, MO, DY, NM, HF, YK, MN, MN, and MI took samples. TS, TT, TA carried out the molecular genetic studies. KT, MW, IH, TA participated in the design of the study. TS, TT, TA performed the statistical analysis. TS wrote the manuscript. TS, TT, TA and IH decided to submit the manuscript for publication. All authors read and approved the final manuscript.

## Pre-publication history

The pre-publication history for this paper can be accessed here:

http://www.biomedcentral.com/1471-230X/9/2/prepub
